# Reactive Oxygen Species-Mediated Control of Mitochondrial Biogenesis

**DOI:** 10.1155/2012/403870

**Published:** 2012-05-30

**Authors:** Edgar D. Yoboue, Anne Devin

**Affiliations:** ^1^CNRS, Institut de Biochimie et Génétique Cellulaires, UMR 5095, 33000 Bordeaux, France; ^2^Institut de Biochimie et Génétique Cellulaires, Université de Bordeaux, UMR 5095, 33000 Bordeaux, France

## Abstract

Mitochondrial biogenesis is a complex process. It necessitates the contribution of both the nuclear and the mitochondrial genomes and therefore crosstalk between the nucleus and mitochondria. It is now well established that cellular mitochondrial content can vary according to a number of stimuli and physiological states in eukaryotes. The knowledge of the actors and signals regulating the mitochondrial biogenesis is thus of high importance. The cellular redox state has been considered for a long time as a key element in the regulation of various processes. In this paper, we report the involvement of the oxidative stress in the regulation of some actors of mitochondrial biogenesis.

## 1. Introduction

**Figure 1:**
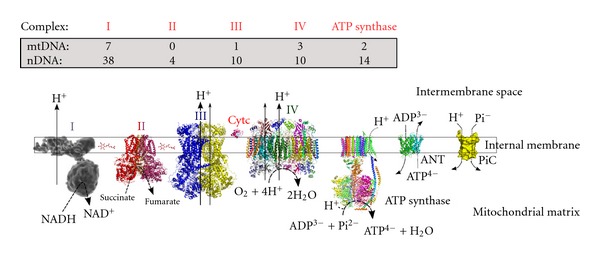
The mammalian oxidative phosphorylations (OXPHOS) system. Depicted are the four respiratory complexes (I–IV), electron carriers coenzyme Q and cytochrome c, the ATP synthase complex, the ADP/ATP carrier (ANC); and the phosphate carrier (PiC). Arrows at complexes I, III, and IV illustrate the proton pumping to the intermembrane space. Indicated are the number of complex subunits encoded by mitochondrial (mtDNA) and nuclear (nDNA) genomes.

**Figure 2:**
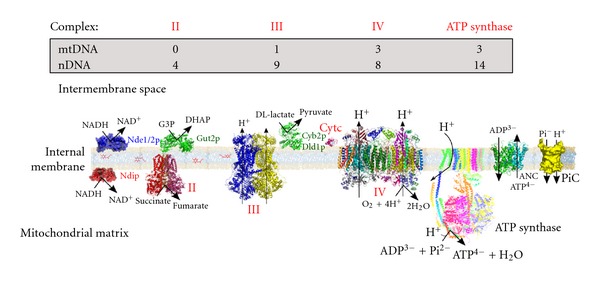
The *Saccharomyces cerevisiae* oxidative phosphorylations (OXPHOS) system. The main differences with the mammalian OXPHOS system are the absence of complex I that is substituted by external and internal NADH dehydrogenases, and the presence of D, L-lactate dehydrogenases, which transfer electrons directly to cytochrome c. Indicated are the number of protein subunits encoded by mitochondrial (mtDNA) and nuclear (nDNA) genomes.

Mitochondria are organelles that have critical functions in eukaryotic cells. Besides their well-known involvement in energy and intermediary metabolism (i.e., ATP synthesis, thermoregulation, heme biosynthesis), mitochondria also play a crucial role in both calcium homeostasis and apoptosis. Mitochondrial dysfunction has been associated with numerous pathologies including neurodegenerative diseases [1], diabetes [2], and aging [3, 4]. ATP synthesis by mitochondria is mostly generated through oxidative phosphorylation (OXPHOS) (Figures 1 and 2). Enzymatic complexes of the mitochondrial respiratory chain couple the oxidation of reducing agents such as NADH and FADH_2_ to proton extrusion toward the intermembrane space. Due to the low proton permeability of the inner mitochondrial membrane, this proton extrusion results in the establishment of an electrochemical potential difference in protons across this membrane. This proton electrochemical potential difference is, in turn, used for ATP synthesis by the F_0_F_1_-ATP synthase complexes.

Mitochondria (and chloroplasts) are unique among eukaryotic extranuclear organelles in that they contain their own genome (mtDNA). In mammalian cells, mtDNA is a circular molecule, which encodes for 13 mRNAs, 22 tRNAs, and 2 rRNAs. All 13 mRNAs encode subunits of the OXPHOS. However, mitochondria are genetically semiautonomous in that they rely strongly on the nuclear genome for their biological function. Indeed, all the remaining mitochondrial proteins, including protein machineries involved in mtDNA replication, transcription, and translation, are encoded by the nuclear genome (Figures 1 and 2).

Consequently, mitochondrial biogenesis is a highly regulated process that involves the coordinated expression of two distinct genomes. This represents an important field of research notably because it has been well established in a wide range of cell types that mitochondrial content within the cell can vary massively depending on the physiological state [5, 6]. For example, a decrease in mitochondrial content has been described in numerous pathologies such as type 2 diabetes [7]. The signals and actors involved in the regulation of mitochondrial biogenesis are thus of high importance. Some of these pathologies are also associated with an oxidative stress, which raises the question of a possible regulation of mitochondrial biogenesis by the cellular redox state that would include ROS levels and glutathione redox state.

In this paper, we focus on some actors of mitochondrial biogenesis—the increase of the mitochondrial enzymatic content—at the transcriptional level in both mammalian cells and the yeast *Saccharomyces cerevisiae *and on their regulation by the cellular redox state.

## 2. Modulation of the Mitochondrial Content in Mammalian Cells

### 2.1. Overview

One of the best illustrations of the variation of the cellular mitochondrial content is the adaptation to energy demand [6]. In the skeletal muscle, in the 1960s, experiments have demonstrated an increase in the number and the size of mitochondria associated with an increase in the activity of mitochondrial marker enzymes in response to exercise [8, 9]. Twenty years later, chronic contractile activity produced by electrical stimulation was shown to increase mRNA levels encoding both nuclear and mitochondrial gene products [10]. These modifications are part of the phenomenon of exercise-induced muscle plasticity [11, 12] and are presumably an adaption in order to adequately match ATP synthesis to ATP consumption by the contractile activity.

Proliferation of mitochondria also occurs during adaptive thermogenesis in brown fat during cold exposure. This proliferation coincides with an increase in the expression of UCP-1, an uncoupling protein that dissipates the proton gradient, leading to an increase in mitochondrial respiration and to heat production [13–15].

### 2.2. Transcription Factors

Nuclear respiratory factors (NRF-1 and NRF-2) were the first identified nuclear transcription factors governing respiratory gene expression in mammalian cells. NRF-1 was discovered when studying the regulation of the cytochrome *c* encoding gene [16, 17]. This protein binds to DNA as a homodimer and functions as a positive regulator of gene transcription. Inactivation of NRF-1 results in early embryonic lethality, pointing out its essential function [18]. NRF-2 was identified by the analysis of the regulation of cytochrome c oxidase (COX) subunits encoding genes [19]. It is a complex of five subunits that shares some target genes with NRF-1. Both NRF-1 and NRF-2 are well known to regulate the transcription of many *respiratory* genes, that is, subunits of complex I, complex II, complex III, COX and ATP synthase, genes encoding proteins involved in mtDNA transcription and replication, as well as genes encoding proteins involved in mitochondrial protein import [17, 20–23]. Peroxisome proliferator-associated receptors (PPAR*α*, PPAR*β*, PPAR*γ*) and ERR*α* are nuclear hormone receptors associated with mitochondrial metabolism. These proteins act as heterodimers and seem to coordinate the expression of genes involved in both fatty acid oxidation and the respiratory chain [24–26].

The PGC-1 family of coactivators (PGC-1*α*, PGC-1*β*, and PRC) plays a central role in the transcriptional regulation of mitochondrial biogenesis. PGC-1*α* was primarily identified as a key actor of adaptative thermogenesis [27]. PGC-1*β* and PRC were discovered through research of sequence similarity to PGC-1*α* [28, 29]. These coactivators act as coordinators of the activity of numerous transcription factors involved in the mitochondrial biogenesis process. Indeed, the PGC-1 proteins, through binding to other transcription factors such as NRF-1, PPARs, and ERR*α*, regulate their activity and are involved in an increase in the expression of transcription factors like NRF-1 and NRF-2 [30]. Overexpression of PGC-1*α* or PGC-1*β* in cultured cells and transgenic mice results in an increase of cellular mitochondrial content [30, 31]. Overexpression in the skeletal muscle induces the conversion to oxidative type muscle fibers [32, 33]. These results strengthen the importance of the PGC-1 family in mitochondrial biogenesis.

### 2.3. Regulation of Mitochondrial Biogenesis by the Oxidative Stress

Mitochondria play an important role in the cellular redox homeostasis due to their main function—that is, oxidation of reduced NADH and FADH_2_—but also through their involvement in ROS metabolism. Indeed, mitochondria are considered as one of the main sites of ROS production in the cell [34]. Considered for a long time as toxic byproducts of oxidative metabolism, ROS are now also considered as signaling molecules, which mediate redox regulation of multiple processes such as cell proliferation, differentiation and apoptosis [34, 35]. Moreover, a regulation of transcription factors involved in mitochondrial biogenesis by oxidative stress has been reported (see the following).

In the late 1990s, an increase in the expression level of mitochondrial proteins mRNAs by oxidative stress was reported. Indeed, the addition of antimycin A—a well-known inhibitor of respiratory chain complex III which increases mitochondrial ROS production—to human fibroblasts, at a concentration partially inhibiting the cellular respiratory, rate led to an increase in cytochrome c1 and cytochrome b mRNA levels [36]. In accordance with this study, another team showed that addition of hydrogen peroxide (H_2_O_2_) to lung fibroblasts led to an increase in the expression of the transcription factor NRF-1, which is involved in mitochondrial biogenesis [37]. Moreover, lipopolysaccharide-induced oxidative damage leads to the upregulation of NRF-1 and NRF-2 and the redox regulation of NRF-1 binding to target promoters is supposed to be mediated through Akt-dependent phosphorylation [38].

St-Pierre et al. [39] have shown that treatment of mouse embryonic cells with H_2_O_2_, increases PGC-1*α* and PGC-1*β* mRNA levels. Previously, several studies showed that PGC-1*α* and PGC-1*β* regulate the expression of genes encoding enzymes involved in the ROS defense system, that is, catalase and superoxide dismutases (SODs) [40–42]. Moreover, PGC-1*α* expression is important for resistance to oxidative damage increase, neurodegeneration, and apoptotic cell death [39, 41]. Thus, those results depict a process where there is a tight link between the biogenesis of a main ROS source production, that is, mitochondria, and the anti-ROS system. Furthermore, PGC-1*α* induction by oxidative stress is partly mediated by binding of the cAMP responsive element binding protein (CREB) in the PGC-1*α* promoter [39].

In skeletal muscle cells, data provided by the Hood team described a ROS-mediated regulation of PGC-1*α* transcription [43]. Indeed, treatment of C_2_C_12_ cells with H_2_O_2_ resulted in an increase in PGC-1*α* mRNA that was prevented by pretreatment of these cells with an antioxidant: N-acetyl-L-cysteine (NAC). In this paper, the induction of PGC-1*α* seemed to depend on the activation of the AMP-activated protein kinase (AMPK) by H_2_O_2_. That activation increased the DNA binding activity of the transcription factor USF1 to the PGC-1*α* promoter, resulting in an increase in PGC-1*α* expression. This work is one of the numerous *in vitro* and *in vivo* studies related to the highly debated question of the relationships between skeletal muscle, oxidative stress, and physical exercise. Since the 1980s, a stimulus role for skeletal muscle adaptation to exercise has been suggested [44]. In recent years, different groups reported that oral administration of antioxidants prevents the exercise-induced adaptation of muscle mitochondria probably through preventing the induction of transcription factors such as PGC-1*α* [45, 46]. Thus, ROS produced during exercise could stimulate mitochondrial biogenesis. However, contradictory results have also been provided about the inhibitory effect of antioxidant addition [47, 48] and this research area is still a strong matter of debate [49, 50].

Diabetes consists in a group of metabolic diseases characterized by defects in the control of glucose and insulin homeostasis, which are a major public health issue. Type 2 diabetes, the most widespread type of diabetes, is known to be associated with alterations in mitochondrial density and mitochondrial dysfunctions in the skeletal muscles of patients [2, 51]. Transcriptomic profile analysis of type 2 diabetes patients revealed a reduced expression level of genes encoding proteins involved in the OXPHOS system [52, 53]. Hyperglycemia is known to increase intracellular ROS levels. This is supposed to occur via several mechanisms [54], one of them being an hyperpolarization of the mitochondrial inner membrane-favorable conditions for ROS production by the mitochondrial respiratory chain [55]—due to impairment in the regulatory capacity of the mitochondrial respiratory chain. Bonnard et al. showed that in hyperglycemic and hyperlipidemic mice, an increase in muscle ROS production is associated with mitochondrial alterations and a decrease in the expression of PGC1-*α* mRNA and some of its target genes. In a model of hyperglycemia-associated oxidative stress (streptozotocin treated mice), NAC treatment restores mitochondrial density and structure [56]. In conclusion, this study suggests that hyperglycemia and hyperlipidemia-induced ROS production in skeletal muscle leads to mitochondrial dysfunction due to a decrease in the expression of PGC1-*α* and its target genes. Although these results seem contradictory if we compare them to the Hood team's, they might be reconciled through the physiological state of the cell. Indeed, downregulation of AMPK activity has been shown in animal models of insulin resistance, high fat feeding, and glucose infusion [57, 58]. Thus, energetic parameters should be considered to interpret the consequences of the oxidative stress on mitochondrial biogenesis.

## 3. Modulation of Mitochondrial Content in the Yeast *Saccharomyces cerevisiae*

### 3.1. Overview

The budding yeast *Saccharomyces cerevisiae* is the unicellular eukaryotic microorganism with the best annotated complete genome sequence. It has been widely used to study molecular mechanisms underlying diverse biological aspects such as mitochondrial functions. Indeed, when yeast cells are grown on nonfermentable carbon source, that is, lactate or ethanol, mitochondria are the unique source of ATP. As stated previously, cells adapt to their energy needs by adjusting their mitochondrial enzymatic content resulting in a capacity to modulate the ATP turnover [6]. In living cells, growth is the result of coupling between substrate catabolism and multiple metabolic processes taking place during net biomass formation and cell maintenance. A crucial parameter for growth description is its yield, that is, the efficiency of the transformation from substrate consumption to biomass formation. When yeast cells are grown on a purely respiratory substrate, biomass generation is entirely connected to substrate oxidation through oxidative phosphorylations and, hence, to oxygen consumption. We have previously shown that, in nonfermentable media, the growth yield is identical regardless of the strain, growth phase, and respiratory substrate used [59]. This homeostasis is the consequence of a strict linear relationship between growth and respiratory rate. Moreover, the oxygen consumption rate was strictly controlled by the cellular content in respiratory chains in such a way that, *in vivo*, the steady state of oxidative phosphorylation was kept constant. The cAMP signaling pathway is now well known to be involved in the regulation of mitochondrial biogenesis, both in mammalian cells and in yeast, even though the molecular mechanisms of this process are not well defined. It has been shown that treatment of human preadipocytes with forskolin, which leads to an overactivation of the cAMP pathway, increased mitochondrial DNA copy number [60]. In yeast, we showed that overactivation of the Ras/cAMP pathway leads to an increase in the cell mitochondrial content [61, 62]. Yeast has three A kinase catalytic subunits, which have greater than 75% identity and are encoded by the TPK (*TPK1, TPK2, *and* TPK3*) genes [63]. Although they are redundant for viability and functions such as glycogen storage regulation, the three A kinases are not redundant for other functions [64–66]. We have shown that in the absence of the yeast protein kinase Tpk3p only, there is a significant decrease in cellular mitochondrial content, when cells are grown in nonfermentable medium [67]. This generates a drastic decrease in cell growth in the Δ*tpk*3 cells versus the wild type cells, since when yeast cells are grown on respiratory substrate, energy transformation processes involve oxidative phosphorylation [59].

### 3.2. The HAP Complex and the Regulation of Mitochondrial Biogenesis by Redox Agents in the Yeast Saccharomyces cerevisiae

**Figure 3:**
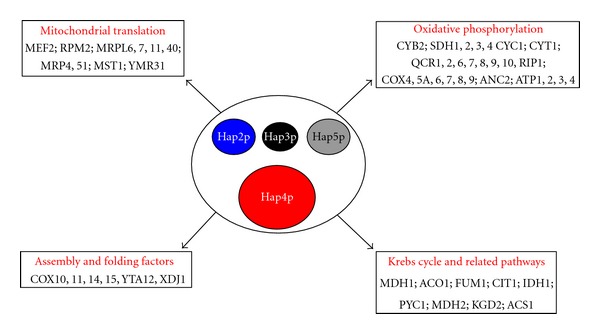
The HAP complex: a master regulator of the mitochondrial biogenesis in the yeast *Saccharomyces cerevisiae*. The four subunits constituting the complex are represented here. Size differences illustrate the difference in the predicted molecular weights of each subunit. The mitochondrial proteins encoding genes regulated by the complex are also indicated. See text for references.

Similarly to what was shown in mammalian cells, the first identification of transcriptional factors regulating mitochondrial biogenesis in *S. cerevisiae* resulted from the study of the regulation of the cytochrome c gene expression (*CYC1*). The master regulator of mitochondrial biogenesis in *S. cerevisiae* is the HAP complex. It is constituted of four subunits: Hap2p, Hap3p, Hap4p, and Hap5p (Figure 3). Subunits 2, 3, and 5 are DNA binding subunits whereas Hap4p is the activator of the complex [68–72]. As illustrated in Figure 3, the HAP complex regulates the expression of many genes encoding proteins involved in mitochondrial functions [73, 74]. In accordance with that key role, the absence of any subunits of the HAP complex leads to a growth defect on nonfermentable medium (i.e., lactate or ethanol). During growth of *Saccharomyces cerevisiae* on fermentable medium (containing high glucose concentration (i.e., 2% (*p*/*v*)), there is a repression of the expression of several genes encoding mitochondrial proteins [75–77]. Under these conditions, it has been shown that overexpression of Hap4p, the activator subunit of the HAP complex, was sufficient to derepress those genes [74, 78]. These results strengthen the main role played by the HAP complex in the regulation of mitochondrial biogenesis. For a long time, the only known signal regulating the HAP complex was the carbon source. Indeed, whereas Hap2p, Hap3p, and Hap5p are constitutively expressed, Hap4p expression is maintained at a very low level during growth on fermentable substrates. Growth on nonfermentable substrates strongly induces Hap4p expression and thus the activity of the HAP complex [71].

As stipulated before, in Δ*tpk3* cells, there is a decrease in mitochondrial content. Moreover, in these cells we have shown that the activity of ROS detoxifying enzymes such as catalase and superoxide dismutases is strongly induced. Mitochondria isolated from *Δtpk3* cells have a high H_2_O_2_ production rate and an elevated level of protein carbonylation [79]. These results led us to hypothesize that the Δ*tpk3* cells were subjected to an oxidative stress. Treatment of *Δtpk3 *cells with an antioxidant such as NAC, as well as Sod1p (superoxide dismutase isoform 1) overexpression, leads to a full restoration of growth, cellular respiratory rates, and mitochondrial content. This clearly indicates that the decrease in mitochondrial content of the *Δtpk3* cells is due to an increase in mitochondrial ROS production. In order to understand the link between ROS and mitochondrial biogenesis, we assessed HAP complex activity in these cells. We were able to show that HAP complex activity was reduced in Δ*tpk3* cells and restored to the wild type level upon antioxidant treatment. Moreover, addition to wild type cells of hydrogen peroxide or antimycin A decreases Hap4p level, the activator subunit of the HAP complex [79]. Thus, ROS regulates mitochondrial biogenesis in the yeast *Saccharomyces cerevisiae* through the regulation of HAP complex activity by decreasing the Hap4p protein level. This work illustrates a mitochondrial control-quality process in which the main transcription factors involved in mitochondrial biogenesis are able to sense a mitochondrial dysfunction and then decrease the biogenesis of these dysfunctional mitochondria.

Those observations are reinforced by our recent study linking the glutathione redox state and the mitochondrial biogenesis. Glutathione is considered as the major redox buffer of the cell because of both its high concentration (1–10 mM) and its low redox potential (*E*_hc_ = −240 mV) [80, 81]. Reduced glutathione (GSH) is a cosubstrate in many ROS detoxifying reactions and protein oxidation repairs (i.e., glutathione peroxidase, glutaredoxins) producing its oxidized form (GSSG). The glutathione reductase, encoded by the *GLR1* gene in the yeast *Saccharomyces cerevisiae*, is a very important enzyme in the glutathione system because it regenerates GSH. The deletion of *GLR1* leads to a decrease in the 2GSH/GSSG ratio. In *Δglr1* cells growing in nonfermentable medium, we observed a very low Hap4p level associated with a low mitochondrial content. The addition of reduced glutathione to *Δglr1* cells induced an increase in this ratio and so resulted in a more reduced cell redox state [82]. Under such conditions, we showed that both Hap4p level and mitochondrial content were restored [83].

In 2010, the transcription factor Hcm1p was identified as another possible regulator of mitochondrial metabolism in the yeast *Saccharomyces cerevisiae* [84]. Indeed, the over-expression of Hcm1p leads to an increase in the cellular respiratory rate and its absence impairs cell growth on ethanol. Interestingly, the nucleus localization of Hcm1p is increased by addition of hydrogen peroxide and Hcm1p increases the expression of antioxidant enzymes such as Sod2p. Thus, Hcm1p could link mitochondrial biogenesis and the ROS detoxification systems. Although the mechanisms clearly explaining the link between Hcm1p—formerly known for its involvement in the regulation of the spindle pole—have not been shown, this result could represent a novel piece to the puzzle of the relationship between the oxidative stress and the regulation of the mitochondrial biogenesis.

## 4. Conclusion

Due to its dependence on two physically distinct genomes, the biogenesis of mitochondria has to be a well-coordinated and regulated process. The identification of nuclear transcription factors regulating the expression of both mitochondrial and nuclear genes encoding for mitochondrial proteins was a great advance in that domain. In the landscape of the regulating signals of the mitochondrial biogenesis, the cellular redox state is becoming an important actor. Mitochondria being one of the main sites of ROS production, and many pathologies being associated with both decrease in mitochondrial content and oxidative stress, these studies naturally raise the question of the crosstalk between the mitochondria and the nucleus. Both decrease and increase of mitochondrial biogenesis in oxidative conditions have been reported. In mammalian cells, the tight link between the regulation of mitochondrial biogenesis and the antioxidant systems has been mechanistically well described. Thus, it seems logic that an increase of the mitochondrial biogenesis by ROS relies on a regulatory system, which is built to *prevent* the cell from the ROS production due to increase of the mitochondrial metabolism. However, in conditions of excessive oxidative stress as in the case of some pathologies and/or severe dysfunction of the mitochondrial respiratory chain, a decrease in mitochondrial biogenesis could be considered as a quality-control process through the decrease of dysfunctional mitochondria by ROS. Because of the key roles played by mitochondria in energy metabolism and many other processes, alternative regulatory pathways relying, for example, on energetic parameters must be taken in consideration. More detailed analyses, notably in regard to the eventual signal specificity according to the nature of the oxidative stress, will certainly be necessary to improve our understanding of the relationships between the oxidative stress and the mitochondrial biogenesis process.
